# Modulation Classification Using Compressed Sensing and Decision Tree–Support Vector Machine in Cognitive Radio System

**DOI:** 10.3390/s20051438

**Published:** 2020-03-06

**Authors:** Xiaoyong Sun, Shaojing Su, Zhen Zuo, Xiaojun Guo, Xiaopeng Tan

**Affiliations:** College of Intelligent Science and Technology, National University of Defense Technology, Changsha 410073, China; sunxiaoyong14@nudt.edu.cn (X.S.); susj-5@163.com (S.S.); jeanakin@nudt.edu.cn (X.G.); tanxiaopeng14@nudt.edu.cn (X.T.)

**Keywords:** modulation classification, high-order cumulant, cyclic spectrum, compressed sensing, decision tree–support vector machine

## Abstract

In this paper, a blind modulation classification method based on compressed sensing using a high-order cumulant and cyclic spectrum combined with the decision tree–support vector machine classifier is proposed to solve the problem of low identification accuracy under single-feature parameters and reduce the performance requirements of the sampling system. Through calculating the fourth-order, eighth-order cumulant and cyclic spectrum feature parameters by breaking through the traditional Nyquist sampling law in the compressed sensing framework, six different cognitive radio signals are effectively classified. Moreover, the influences of symbol length and compression ratio on the classification accuracy are simulated and the classification performance is improved, which achieves the purpose of identifying more signals when fewer feature parameters are used. The results indicate that accurate and effective modulation classification can be achieved, which provides the theoretical basis and technical accumulation for the field of optical-fiber signal detection.

## 1. Introduction

As one of the booming communication technologies in the information era, modulation classification (MC) technology [1] has a very important application value in the field of wireless communication. For example, it can play an important role in communication investigation, electronic countermeasures, signal authentication, interference identification, spectrum management, etc. At present, the wireless communication network has maintained a steady and rapid development trend, the network construction is increasingly integrated, and network applications are everywhere. At the same time, the inherent contradiction between the centralized static network and the dynamic change of the environment also causes serious problems like the low utilization of spectrum resources in the wireless communication network. Therefore, cognitive radio (CR) technology [2,3,4,5] is proposed and considered as a promising technology to solve these problems. MC plays an important role in CR based on spectrum sensing and feature analysis.

Cognitive radio has been widely accepted as a new technology in the field of wireless communication in the new era. In the cognitive radio network (CRN), in order to avoid interference with the transmission of the primary users, it is essential to accurately sense the presence for any contemporaneous transmission of the primary users in the observed spectrum [6]. The primary user signal error detection will cause the secondary user to waste the spectrum opportunity. Noise, shadow and multipath fading lead to a serious degradation of signal characteristics in conventional wireless communication scenarios. This makes signal detection very difficult in a low signal-to-noise ratio (SNR) environment [7,8]. In addition, because the primary user (authorized user) and the cognitive user (unauthorized user) cannot communicate with each other, accurate MC can not only avoid mutual interference between them, but also provide the multi-dimensional spectrum information of the surrounding wireless environment, which helps to improve the inefficient use of spectrum resources in the CRN. With the different modulation parameters and methods used in the wide-band communication signal, MC has gradually been studied in depth and has become one of the main methods of signal recognition and classification.

MC has been playing an important role in the field of wireless communication for a long time, especially in dynamic spectrum management and interference recognition. A variety of methods and classifiers have been proposed in the literature, but most of them only identify a few modulation formats, such as low-order modulation format, or require some knowledge of parameters of the signal. MCs of a CR system are roughly divided into 4 categories: (a) Multiple quadrature amplitude modulation (MQAM) and multiple phase shift keying (MPSK) signals are classified based on signal envelope variance and wavelet transform, but the recognition rate is low at low SNR [9,10]; (b) artificial neural networks (ANN) based on machine-learning algorithms for automatic signal type recognition, which requires the most appropriate ANN and will lead to an increase in calculation time and risk of over-fitting [11,12]; (c) identification from higher-order cumulant (HOC) using fourth-order cumulant, which cannot identify some signals with the same fourth-order cumulant [13,14]; (d) feature parameters are extracted from the time domain, frequency domain and power spectrum of signals to classify and identify a modulation signal, but some feature parameter extraction processes are complex and easily interfered with by noise [15,16,17]. The proposed method mainly focuses on the recognition of single-feature parameters, and most classifiers adopted an increase the complexity of the system.

In this paper, we propose a new modulation classification method that combines high-order cumulants and cyclic spectrum feature extraction methods with a decision tree–support vector machine (DT–SVM) classifier. In the feature extraction phase, the compressed sensing (CS) method is used to obtain the compressed sample size of the feature parameters, and the influence of key factors on the classification accuracy in the modulation classification process is analyzed. CS is a signal processing technique called “sampling compression combo”. The CS method can map signals from high-dimensional space to low-dimensional space through a small number of observations (non-adaptive linear projection) of sparse signals, and maintain the original structure of the signal [18]. The sparse signal reconstruction is actually reconstructing the original signal from the signal observations with high probability by solving the non-linear optimization problem, which breaks through the limitations of the traditional Shannon–Nyquist sampling theorem and solves the performance requirements of a sampling system when processing cognitive radio signal. It also relieves the pressure of storage, transmission and processing for large amounts of the traditional sampled data. The combination of HOC and the cyclic spectrum can distinguish the same cumulant of different signals, and achieve the MC through the DT–SVM classifier. Combining the advantages of HOC and cyclic spectrum features, the algorithm directly obtains the compressed values of feature parameters through the CS theory, and analyzes the influence of symbol length and compression ratio on the recognition accuracy. The simulation results show that the algorithm has a better classification performance in low SNR and the validity of the method is verified.

The rest of this paper is structured as follows. Section 2 introduces the feature extraction method and its characteristics in detail. Section 3 introduces the compression sampling values of feature parameters obtained by combining the compression sensing theory. Section 4 describes the structure of the decision tree–support vector machine classifier. In Section 5, some simulation results are presented. Finally, Section 6 sums up the conclusions.

## 2. Feature Extraction 

### 2.1. Feature Extraction Based on Higher-Order Cumulant (HOC) 

For wireless channel model, we studied the property of HOC and the insensitivity of its second-order terms to Gaussian noise, the kth-order cumulant $C_{k,n}(m_{1},m_{2},\cdot \cdot \cdot ,m_{k-1})$ of a complex-valued stationary random process x(t), can be defined as:

(1) $$C_{k,n}(m_{1},m_{2},\cdot \cdot \cdot ,m_{k-1})=cum(x(t),x(t+m_{1}),\cdot \cdot \cdot ,x(t+m_{k-1}))$$

where $x(t+m_{k})$ denotes a function of different time delays and regardless of t, cum(•) means taking the cumulant. Therefore, its fourth-order cumulant is:

(2) $$\begin{array}{ll} C_{4,n}(m_{1},m_{2},\cdot \cdot \cdot ,m_{3})= & E[x(n)x(n+m_{1})x(n+m_{2})x(n+m_{3})]-C_{2,n}(m_{1})C_{2,n}(m_{2}-m_{3})\\ & -C_{2,n}(m_{2})C_{2,n}(m_{3}-m_{1})-C_{2,n}(m_{3})C_{2,n}(m_{1}-m_{2}) \end{array}$$

Based on the above theory, the fourth-order, sixth-order and eight-order cumulants of the zero-mean x(t), are shown as:

(3) $$\begin{array}{l} C_{4,0}=cum(x,x,x,x)=M_{4,0}-3{M_{2,0}}^{2}\\ C_{4,1}=cum(x,x,x,x^{*})=M_{4,1}-3M_{2,1}M_{2,0}\\ C_{4,2}=cum(x,x,x^{*},x^{*})=M_{4,2}-{\left|M_{2,0}\right|}^{2}-2{M_{2,1}}^{2}\\ C_{6,0}=cum(x,x,x,x,x,x)=M_{6,0}-15M_{4,0}M_{2,0}+30{M_{2,0}}^{3}\\ C_{6,3}=cum(x,x,x,x^{*},x^{*},x^{*})=M_{6,3}-9C_{4,2}C_{2,1}-6{C_{2,1}}^{3}\\ C_{8,0}=cum(x,x,x,x,x,x,x,x)=M_{8,0}-28M_{6,0}C_{2,0}-35{M_{4,0}}^{2}+420M_{4,0}{M_{2,0}}^{2}-630{M_{2,0}}^{4} \end{array}$$

where $M_{pq}=E[x{\left(t\right)}^{p-q}x^{*}{\left(t\right)}^{q}]$ denotes the pth-order mixing moment [19]. 

In the practical application of MC, we need to estimate the HOC value of the signal from the received symbol sequence in the shortest possible time. Sample estimations of the correlations are given by:

(4) $$\begin{array}{l} C_{4,0}=\frac{1}{N}\sum_{n=1}^{N}{\left(x(t)\right)}^{4}-3C_{2,0}^{2}\\ \;\cdot \cdot \cdot \cdot \cdot \\ C_{8,0}=\frac{1}{N}\sum_{n=1}^{N}{\left(x(t)\right)}^{8}-28C_{2,0}\frac{1}{N}\sum_{n=1}^{N}{\left(x(t)\right)}^{6}-35M_{4,0}^{2}+420M_{4,0}M_{2,0}^{2}-630M_{2,0}^{2} \end{array}$$

Substituting the estimated values into Equation (4), we can obtain all of the features for the considered six wireless signal types. Table 1 shows some of these features for a number of these signals. These values are computed under the constraint of unit variance in noise free conditions. It can be seen that by computing of these values, we can classify the wireless signal types.

Table 1 shows that OOK (on-off keying), DPSK (differential phase shift keying), QPSK (quadrature phase shift keying), OQPSK (offset quadrature phase shift keying) have the same theoretical values of HOC. In addition, 16QAM (16 quadrature amplitude modulation) and 64QAM (64 quadrature amplitude modulation) have similar HOC values. Therefore, we can define a feature parameter $T1=\left|C_{8,0}\right|/\left|C_{4,0}\right|$ that is calculated in Table 2 and divides signals into three categories including (OOK, DPSK), (QPSK, OQPSK) and (16QAM, 64QAM). It is worth noting that the absolute value and ratio form are used to eliminate the effect of phase jitter and amplitude [20].

Owing to the difference between the phase jump rules of QPSK and OQPSK, the sampling sequence of both can be performed with a differential operation, i.e.,

(5) $$\Delta x(t)=x(t+1)-x(t)=(a_{t+1}-a_{t})\exp[j(2\pi f_{c}+\Delta \theta_{c})]$$

where x(t) denotes the signals of QPSK and OQPSK, $a_{k}$ is the transmitted symbol sequences, $f_{c}$ denotes the carrier frequency and $\theta_{c}$ denotes the phase jitter. For the sake of discussion, we assume that $f_{c}$ and $\theta_{c}$ have been completed timing synchronization. The values of HOC under difference operation are calculated in Table 3. Then we define another feature parameter $T2={\left|C_{d8,0}\right|/\left|C_{d4,0}\right|}^{2}$ is calculated in Table 4 to classify QPSK and OQPSK, where $C_{d8,0}$ and $C_{d4,0}$ represent the cumulants after differential operation.

### 2.2. Feature Extraction Based on Cyclic Spectrum

Since the T1 of (OOK, DPSK) and (16QAM, 64QAM) are the same or similar, a cyclic spectral density function for noise suppression is proposed for identification. Assuming x(t) is the cyclostationary signal, and its mean value and autocorrelation function are periodic with $T_{0}$ shown as:

(6) $$m_{x}(t+T_{0})=m_{x}(t)$$

(7) $$R_{x}(t+T_{0}+\frac{\tau }{2},t+T_{0}-\frac{\tau }{2})=R_{x}(t+\frac{\tau }{2},t-\frac{\tau }{2})$$

where τ is the delay variable. Because the autocorrelation function has periodicity, its Fourier series can be written as:

(8) $$R_{x\alpha }(t+\frac{\tau }{2},t-\frac{\tau }{2})=\sum_{\alpha }R_{x\alpha }(\tau )e^{j2\pi x}$$

where α stands for the frequency corresponding to the instantaneous autocorrelation and is often called the cyclic frequency. In addition, $R_{x\alpha }$ is the coefficient of the Fourier series which is given by:

(9) $$R_{x\alpha }(\tau )=\frac{1}{T_{0}}\int_{-\frac{T_{0}}{2}}^{\frac{T_{0}}{2}}R_{x\alpha }(t+\frac{\tau }{2},t-\frac{\tau }{2})e^{-j2\pi \alpha t}dt$$

The Fourier transform of the cyclic autocorrelation function can be written as:

(10) $$S_{x\alpha }(f)≜\int_{-\infty }^{\infty }R_{x\alpha }(\tau )e^{-j2\pi ft}d\tau$$

where $S_{x\alpha }(f)$ is called power spectral density and f is the spectral frequency.

The $R_{x\alpha }(\tau )$ can be seen as the cross-correlation of two complex frequency shift components u(t) and v(t) of x(t), i.e.,

(11) $$R_{x\alpha }(\tau )=R_{uv\alpha }(\tau )=\frac{1}{T_{0}}\int_{-\frac{T_{0}}{2}}^{\frac{T_{0}}{2}}u(t+\frac{\tau }{2})v^{*}(t-\frac{\tau }{2})dt.$$

where $u(t)=x(t)e^{j2\pi \alpha t}$, $v(t)=x(t)e^{-j2\pi \alpha t}$.

From Equation (10) we can obtain $S_{x\alpha }(f)=S_{uv\alpha }(f)$. Through the cross-spectrum analysis, we can obtain:

(12) $$S_{x\alpha }(f)≜\underset{T_{0}\to \infty }{\lim}\underset{\Delta t\to \infty }{\lim}S_{uvT_{0}}{\left(f\right)}_{\Delta t}=\underset{T_{0}\to \infty }{\lim}\underset{\Delta t\to \infty }{\lim}\frac{1}{\Delta t}\int_{-\frac{\Delta t}{2}}^{\frac{\Delta t}{2}}S_{X_{T_{0}}\alpha }(t,f)dt$$

(13) $$S_{X_{T_{0}}\alpha }(t,f)=\frac{1}{T_{0}}X_{T_{o}}(t,f+\frac{\alpha }{2})X_{T}^{*}(t,f+\frac{\alpha }{2})$$

(14) $$X_{T_{0}}(t,f+\frac{\alpha }{2})=\int_{t-\frac{T_{0}}{2}}^{t+\frac{T_{0}}{2}}x(u)e^{-j2\pi fu}du$$

where Equation (12) is used to estimate the cyclic spectral density, Equation (13) is the cyclic periodic diagram, Equation (14) is the short-time Fourier transform (STFT) formula, Δt is the length of received data, $T_{0}$ is the window length for the STFT, and ${\left(•\right)}^{*}$ is the complex conjugate.

According to the insensitivity of the cyclic spectrum to noise and the above theory, the characteristic parameter $T3=\max(S_{x\alpha })$ is defined to distinguish the signal set of (OOK, DPSK) and (16QAM, 64QAM).

## 3. Compressed Values of Feature Parameters Based on Compressed Sensing

### 3.1. Compressed Value of HOC

CS techniques perform successfully whenever applied to so-called compressible and/or K-sparse signals, i.e., signals that can be represented by K≪N significant coefficients over an N-dimensional basis [21,22]. The K-sparse signal $s(t)\in {\mathbb{R}}^{N}$ of dimension N is accomplished by computing a measurement vector $y(t)\in {\mathbb{R}}^{M}$ that consists of M≪N linear projections of the vector s(t). The compression sampling rate $f_{cs}=(M/N)f_{s}$, where $f_{s}$ is traditional sampling rate and δ=M/N∈(0,1) is called the compression ratio. The linear compression sampling process can be described as:

(15) y=Φs+n

where Φ represents a M×N matrix, usually over the field of real numbers. It is noted that the measurement matrix Φ is a random matrix satisfying the restricted isometry property (RIP) [23], and its form is various, such as Gaussian matrix [24] and local Hadamard matrix [25].

According to the theory of feature extraction and compression sensing, the linear square compression sampling process can be defined as:

(16) $${〚x〛}^{2}=\Phi {〚s〛}^{2}$$

where 〚•〛 represents the product operation of the corresponding elements between the vectors. From Equation (15), we can through CS to simplify the reconstruction.

First, we define a relationship between the autocorrelation matrix $R_{{〚x〛}^{2}}$ and $R_{{〚s〛}^{2}}$:

(17) $$R_{{〚x〛}^{2}}={〚x〛}^{2}{\left({〚x〛}^{2}\right)}^{T}=(\Phi {〚s〛}^{2}){\left(\Phi {〚s〛}^{2}\right)}^{T}=\Phi R_{{〚s〛}^{2}}\Phi^{T}$$

Second, we obtain a relationship between ${〚x〛}^{2}$ and $R_{{〚x〛}^{2}}$:

(18) $${〚x〛}^{2}=P_{{〚x〛}^{2}}vec(R_{{〚x〛}^{2}})$$

where $vec(AXB)=(B^{T}⊗A)vec(X)$ (⊗ denotes Kronecker product) and $P_{{〚x〛}^{2}}\in {\left\{0,1\right\}}^{n\times n^{2}}$ that maps the linearly products to the vectorized counterparts ${〚x〛}^{2}$ and $R_{{〚x〛}^{2}}$ [26].

According to Equation (4), the compressed value of fourth-order cumulant can be defined as:

(19) $$C_{(4,0)\alpha }=F{〚s〛}^{4}-3{〚F{〚s〛}^{2}〛}^{2}$$

where $F=\frac{1}{N}{\left[\exp(-j2\pi \alpha n/N)\right]}_{\left(\alpha ,n\right)}\in {\mathbb{R}}^{M_{\alpha }\times N}$ is the discrete Fourier transform (DFT) matrix.

Therefore, the linear representation process of the vector-form $C_{(4,0)\delta }$ is shown as:

(20) $$\begin{array}{ll} C_{(4,0)\delta } & =FP_{s}vec(R_{{〚s〛}^{2}})-3P_{Fs}vec(R_{F{〚s〛}^{2}})=FP_{s}vec(R_{{〚s〛}^{2}})-3P_{Fs}vec(FR_{{〚s〛}^{2}}F^{T})\\ & =[FP_{s}-3P_{Fs}(F⊗F)]vec(R_{{〚s〛}^{2}})=[FP_{s}-3P_{Fs}(F⊗F)]{\left[P_{{〚x〛}^{2}}(\Phi ⊗\Phi )\right]}^{†}{〚x〛}^{2} \end{array}$$

where ${\left[•\right]}^{†}$ stands for the pseudo-inverse operation.

Finally, by deriving the linear compressed sampling process of the fourth-order cumulant, we can obtain the compressed value of the eighth-order as follows:

(21) $$\begin{array}{ll} C_{(8,0)\delta }= & \{FP_{s}[(P_{s}-28P_{s}^{1/2}P_{Fs}^{1/2}{\left(F⊗F\right)}^{1/2}-35FP_{s}+420P_{Fs}(F⊗F)]-\;\\ & 630P_{Fs}^{2}{\left(F⊗F\right)}^{2}\}•{\left[P_{{〚x〛}^{2}}(\Phi ⊗\Phi )\right]}^{†}{〚x〛}^{2} \end{array}$$

Therefore, the first and second characteristic parameters after CS is $T1=\left|C_{(8,0)\delta }\right|/\left|C_{(4,0)\delta }\right|$ and $T2={\left|C_{d(8,0)\delta }\right|/\left|C_{d(4,0)\delta }\right|}^{2}$.

### 3.2. Compressed Value of Cyclic Spectrum

It can be seen from Equations (10) and (16) that there is no direct linear relationship between the compressed sampled value x in the time domain and the cyclic spectrum $S_{x\alpha }$, so the existing reconstruction algorithm cannot be used to implement the cyclic spectrum estimation. It is necessary to use some explicit linear relations between the second-order statistic to derive its transformation, and indirectly establish the linear relationship between them, so as to use the existing reconstruction algorithm to complete the estimation of the cyclic spectrum [27].

In order to obtain the compressed values of the cyclic spectrum, we first need to obtain the relationship between the cyclic spectrum matrix $S_{s\delta }$ and the cyclic autocorrelation matrix $R_{s\delta }(u,v)$. Let $R_{s\delta }(u,v)$ denote the form of the time-varying covariance matrix R. When x is a real value, R is a symmetric semi-positive definite matrix. For the convenience of calculation, we convert it into an auxiliary covariance matrix:

(22) $$R=\left[\begin{array}{ccccc} R_{s\delta }(0,0) & R_{s\delta }(0,1) & R_{s\delta }(0,2) & \cdots & R_{s\delta }(0,N-1)\\ R_{s\delta }(1,0) & R_{s\delta }(1,1) & R_{s\delta }(1,2) & \cdots & 0\\ R_{s\delta }(2,0) & R_{s\delta }(2,1) & R_{s\delta }(2,2) & \cdots & 0\\ \cdots & \cdots & \cdots & \cdots & \cdots \\ R_{s\delta }(N-1,0) & 0 & 0 & \cdots & 0 \end{array}\right]$$

where N is the sampling point.

The matrix R contains all the elements in the $R_{s\delta }$ vector except for the zero elements. The relationship between $R_{s\delta }$ and R can be expressed as:

(23) $$vec\left\{R\right\}=H_{N}R_{s\delta }$$

where $H_{N}\in {\left\{0,1\right\}}^{N^{2}\times (N(N+1)/2)}$, vec{•} means the vectorization operation, and the $R_{s\delta }$ vector is mapped to vec{R}.

So the relationship between the cyclic spectrum matrix and the cyclic autocorrelation matrix is shown as:

(24) $$\begin{array}{l} R_{s\delta }=\sum_{v=0}^{N-1}G_{v}RD_{v}\\ S_{s\delta }=R_{s\delta }F \end{array}$$

where $G_{n}={\left[\frac{1}{N}\exp(-j\frac{2\pi }{N}a(n+\frac{v}{2}))\right]}_{\left(a,n\right)}\in {\mathbb{R}}^{N\times N}$, $F={\left[\exp(-j2\pi vb/N)\right]}_{\left(v,b\right)}$ is the N-point DFT matrix and $D_{v}$ is an N×N matrix with only its (v,v)th diagonal element being 1 and all other elements being 0. In addition, n and v are time delay, a,b∈[0,N−1] denotes digital cyclic frequency and α=fa/N stands for the cyclic frequency.

The time-varying covariance matrix $R_{x}=E\left\{x_{m}x_{m}^{T}\right\}$ of the compressed value x is also a symmetric semi-definite matrix, which can be rearranged into a vector $R_{x\delta }$ of length M(M+1)/2 to represent as:

(25) $$\begin{array}{ll} R_{x\delta }= & \left[R_{x\delta }(0,0),R_{x\delta }(1,0),\cdots ,R_{x\delta }(M-1,0\right)\\ & R_{x\delta }(0,1),R_{x\delta }(1,1),\cdots ,R_{x\delta }(M-2,1)\\ & R_{x\delta }(0,M-1){]}^{T} \end{array}$$

Through the linear formula conversion, we can define two projection matrices $P_{m}\in {\left\{0,1\right\}}^{N^{2}\times (N(N+1)/2)}$ and $Q_{m}\in {\left\{0,1/2,1\right\}}^{(M(M+1)/2)\times M^{2}}$ map the entries of x, s to those in $vec(R_{x})$ and $vec(R_{s})$, it can be shown that:

(26) $$\begin{array}{l} vec\left\{R_{x}\right\}=P_{m}x\\ s=Q_{m}vec\left\{R_{s}\right\} \end{array}$$

where $P_{m}$ and $Q_{m}$ are special mapping matrices.

Because of x(t)=Φs(t), we can obtain:

(27) $$x=Q_{m}vec(R_{x})=Q_{m}(\Phi ⊗\Phi )vec(R_{s})=Q_{m}(\Phi ⊗\Phi )P_{m}s=\Theta s$$

where $\Theta =Q_{m}(\Phi ⊗\Phi )P_{m}\in {\mathbb{R}}^{\frac{M(M+1)}{2}\times \frac{N(N+1)}{2}}$.

Following the equation (24), we can obtain $vec(R_{s\delta })$ is:

(28) $$vec(R_{s\delta })=\sum_{v=0}^{N-1}\left(G_{v}^{T}⊗D_{v})vec(R_{s}\right)=\Omega s$$

where $\Omega =\sum_{v=0}^{N-1}(g_{v}^{T}⊗D_{v})P_{m}\in {\mathbb{R}}^{N^{2}\times (N(N+1)/2)}$.

Through the Equations (24), (27) and (28), we can derive the measurement vector x as a linear function of the vector-form cyclic spectrum $S_{s\delta }$ as:

(29) $$S_{s\delta }=\;\Xi \Omega \Theta^{†}x$$

where $\Xi ={\left(F^{-T}⊗I_{N}\right)}^{-1}$, $I_{N}$ is the N dimension unit matrix. Therefore, the third characteristic parameter after CS is $T3=\max(S_{s\delta })$.

## 4. The Structural Process of Decision Tree–Support Vector Machine Classifier 

### 4.1. The Principle of Support Vector Machine

Support vector machine (SVM) is based on the principle of structural risk minimization [28,29,30]. Its final solution can be transformed into a quadratic convex programming problem with linear constraints. There is no local minimum problem. By introducing the kernel function, the linear SVM can be simply extended to the non-linear SVM, and there is almost no additional computation for high-dimensional samples.

**Figure 1:**
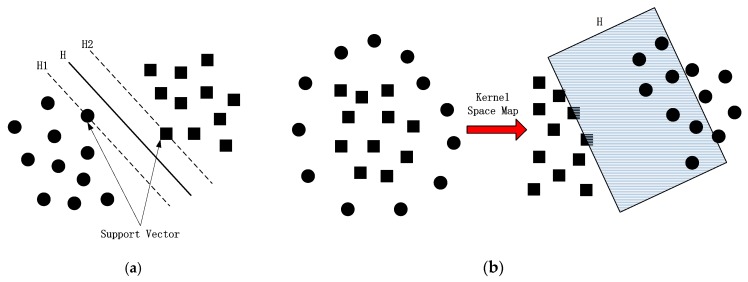
Linear (**a**) and non-linear (**b**) classification of support vector machine (SVM).

The main idea can be seen from Figure 1 that for a linear separable case, the idea of maximizing the classification boundary is used to seek the optimal hyperplane H, while H1 and H2 are hyperplanes passing through the closest sample to the H and parallel to h, respectively, and the distance between them is called the classification interval. In the case of linear indivisibility, the linear indivisible samples in the low-dimensional input space are transformed into the high-dimensional feature space by the non-linear mapping algorithm, so that they can be linearly separable, in order that the high-dimensional feature space can be solved by the linear analysis method.

Suppose that the training set is $\left\{(x_{i},x_{i}),i=1,2,\ldots ,L\right\}$ and the expected output is $y_{i}\in \left\{+1,-1\right\}$, where +1 and −1 represent two kinds of class representation respectively. If $x_{i}\in R^{n}$ belongs to the first category, the corresponding output is $y_{i}=+1$; if it belongs to the second category, the corresponding output is $y_{i}=-1$. The linear separability of the problem shows that there is a hyper-plane (w∗x)+b=0, which makes the positive and negative inputs of the training points located on both sides of the hyper-plane, respectively. When the training sets are not completely linearly separable, we can introduce the relaxation variable $\xi_{i}\geq 0\;i=1,2,\ldots ,L$, then the objective function is transformed into:

(30) $$\begin{array}{l} \min\;\phi (w)=\frac{1}{2}{\left\|w\right\|}^{2}+C\sum_{i=1}^{L}\xi_{i}\\ s.t.\;y_{i}\left[(w\cdot x_{i})+b\right]\geq 1-\xi_{i}\\ \xi_{i}\geq 0\;i=1,2,\ldots ,L \end{array}$$

where C≥0 is the penalty parameter. A larger C indicates a larger penalty for misclassification and is the only parameter that can be adjusted in the algorithm. By choosing the proper kernel function $K(x,x^{T})$ and using the Lagrange multiplier method to solve Equation (30), the corresponding dual problem can be obtained as follows:

(31) $$\begin{array}{l} \underset{\alpha }{\min}\frac{1}{2}\sum_{i=1}^{L}\sum_{j=1}^{L}y_{i}y_{j}\alpha_{i}\alpha_{j}K(x_{i},x_{j})-\sum_{j=1}^{L}\alpha_{j}\\ s.t.\;\sum_{i=1}^{L}y_{i}\alpha_{i}=0\\ \;0\leq \alpha_{i}\leq C\;i=1,2,\ldots ,L \end{array}$$

Equation (31) obtains the optimal solution $\alpha^{*}=(\alpha_{1}^{*},\alpha_{2}^{*},\ldots ,\alpha_{L}^{*})$ and selects a positive component $0\leq \alpha_{j}^{*}\leq C$ of $\alpha^{*}$, and calculates $b^{*}=y_{j}-\sum_{i=1}^{L}y_{i}\alpha_{i}^{*}K(x_{i},x_{j})$ accordingly. Finally, the policy function $f(x)=\mathrm{sgn}(\sum_{i=1}^{L}y_{i}\alpha_{i}^{*}K(x_{i},x_{j})+b^{*})$ is obtained.

### 4.2. The Structure of Decision Tree–Support Vector Machine Classifier

**Figure 2:**
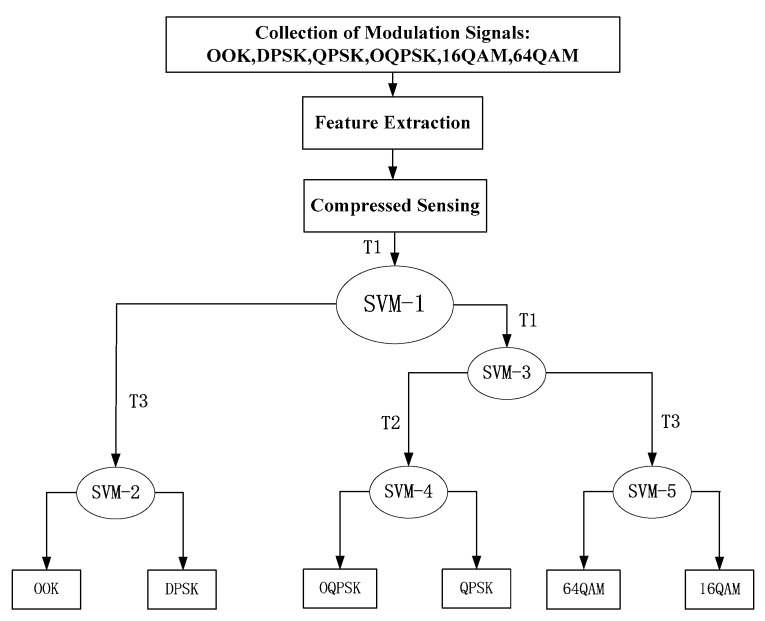
The identification process of decision-tree (DT) classifier.

Through the above calculation and analysis of the compressed values of the feature parameters based on CS, the feature parameters are input into the decision tree–support vector machine (DT–SVM) structure with efficient calculation to realize signal classification. Adding support vector machine (SVM) to every node of the decision tree can comprehensively utilize the efficient computing power of the decision tree structure and the high classification performance of the SVM to achieve the high-precision classification of MC. In particular, for the K-class classification problem, the method of DT–SVM only needs to construct K-1 SVM sub classifier. The classification process of decision tree is shown in Figure 2.

**Figure 3:**
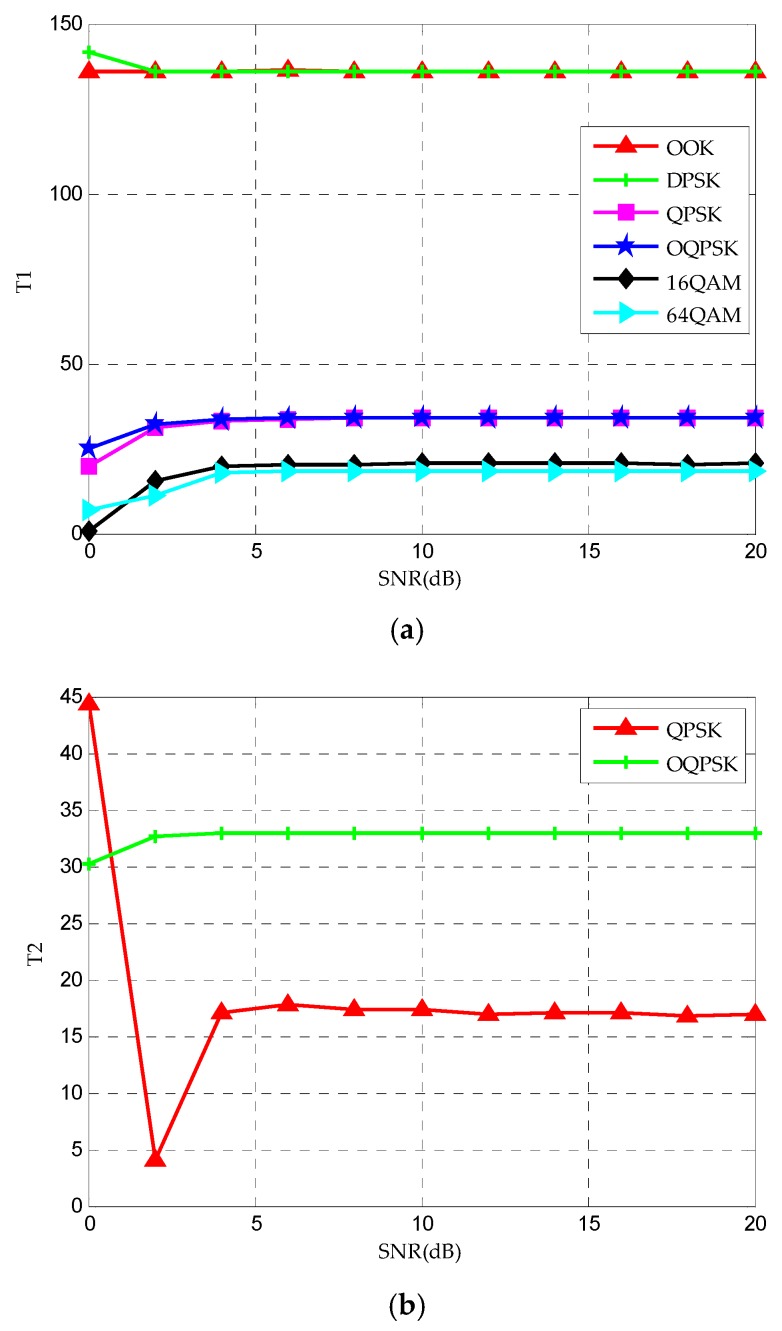
The values of feature parameters (**a**) T1 and (**b**) T2 under different signal-to-noise ratios (SNRs).

**Figure 4:**
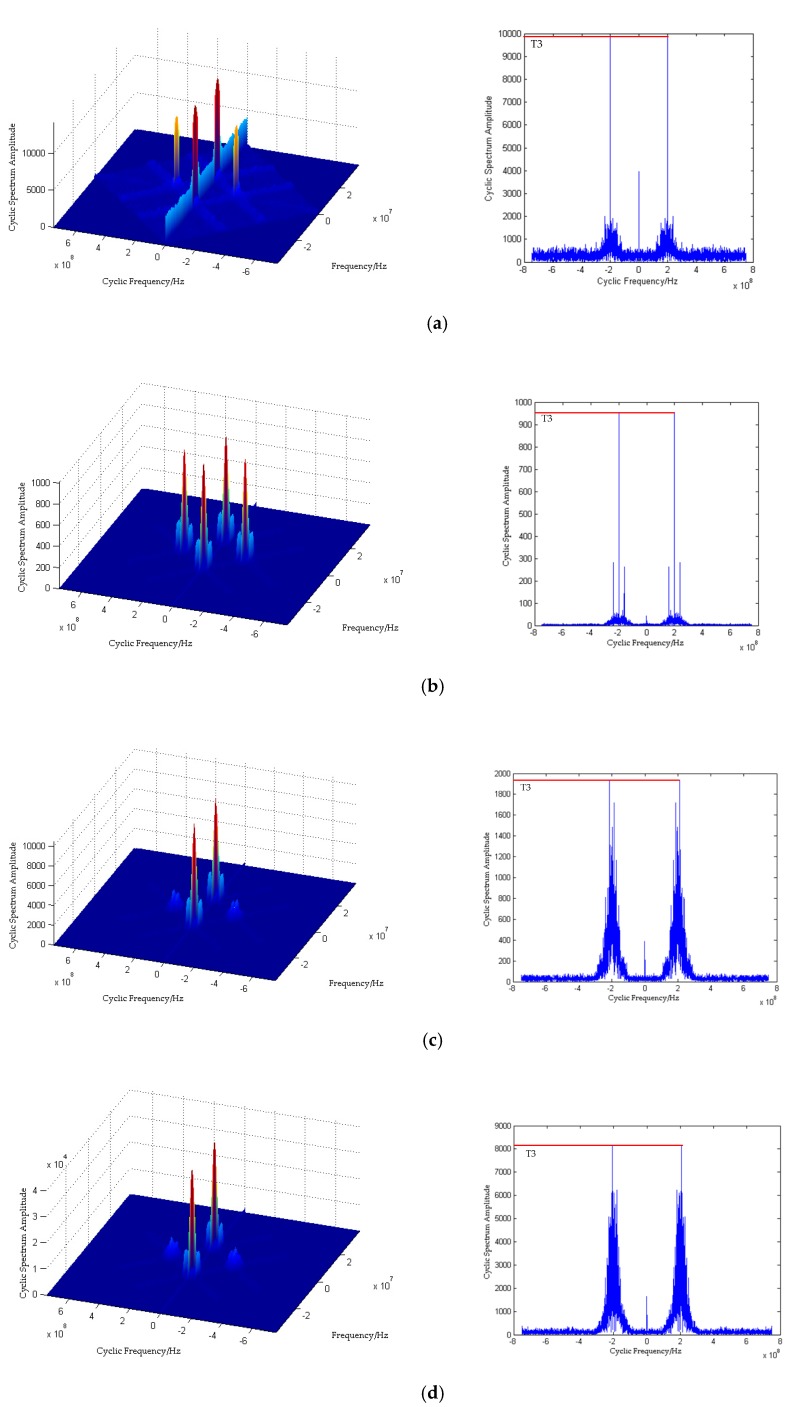
Cyclic spectrum and cross-sectional diagrams of (**a**) OOK (on-off keying), (**b**) DPSK (differential phase shift keying), (**c**) 16QAM (16 quadrature amplitude modulation) and (**d**) 64QAM (64 quadrature amplitude modulation).

Using the three features in Figure 1 to complete the MC, the specific steps of the process are as follows:

(1) Three feature vectors are obtained from six kinds of wireless modulation signal data through feature extraction module;
(2) The feature vector is input into the compression-sensing module to obtain the respective compression sampling values, as shown in Figure 3 and Figure 4;
(3) Six kinds of wireless signals are roughly classified by T1. The (OOK, DPSK) signals can be separated by SVM-1, and the remaining signals are classified into one class;
(4) For (OOK, DPSK) signals, SVM-2 and T3 are used to realize classification;
(5) By SVM-3 and T1, the residual signals can be divided into two categories: (QPSK, OQPSK) and (16QAM, 64QAM);
(6) The T2 after differential operation and SVM-4 are used to classify QPSK and OQPSK;
(7) Finally, the classification of 16QAM and 64QAM signals is realized by the T3 and SVM-5.

It can be seen from Figure 3a that the compressed value of the feature parameter T1 tends to be stable with the increase of SNR and conforms to the theoretical value. In addition, it is obvious from the figure that T1 can classify the six signals into three categories includes (OOK, DPSK), (QPSK, OQPSK) and (16QAM, 64QAM). Similarly, it can be seen from Figure 3b that the feature parameter T2 after difference can distinguish QPSK from OQPSK. The circular spectrum and the cross-sectional diagrams of cycle frequency of OOK, DPSK, 16QAM and 64QAM are shown in Figure 4. It can be seen from Figure 4 that the maximum value of the cyclic spectrum of different signals is different, so the remaining signals can be distinguished by T3.

## 5. Simulation Results and Discussion

For signal modulation classification task, the modulation set is {OOK, DPSK, QPSK, OQPSK, 16QAM, 64QAM}. In this simulation process, all modulation signals adopt the same modulation parameters, that is, the carrier frequency is 100 kHz, the symbol rate is 40 kbps, and the sampling frequency is 800kHz. For each kind of modulation signal, the simulation generates 500 characteristic samples under different SNR (SNR from −5 dB to 15 dB, interval 5 dB). The K-fold cross validation (K-CV) method is used to evaluate the generalization ability of the model, which can not only improve the data utilization, but also solve the over fitting problem to a certain extent, so as to select the model. In this paper, K is chosen as 10. The basic principle of K-CV is to divide the training data set into K equal subsets. Each time, K-1 data are used as training data, and other data are used as test data. In this way, we repeat K times, estimate the expected generalization error according to the mean square error (MSE) average value after K times iteration, and finally select a group of optimal parameters.

**Figure 5:**
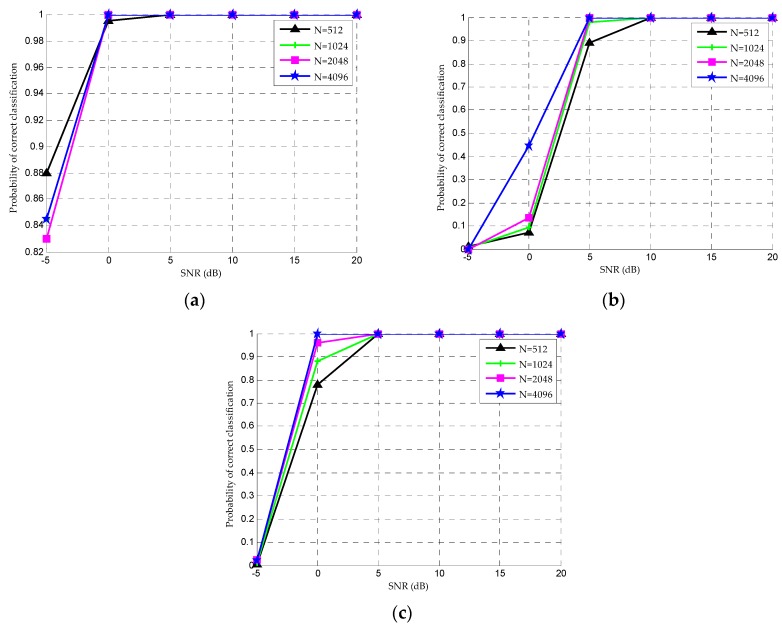
Correct classification rate with different symbol length for (**a**) OOK, (**b**) QPSK and (**c**) 16QAM under different SNRs.

To evaluate the influence of different symbol lengths on the classification performance, we select the symbol length N = 512, 1024, 2048, 4096 for the OOK, QPSK and 16QAM signals (as examples), respectively, to analyze the classification accuracy in Figure 5. As can be seen from Figure 5, with the increase of symbol length, the trend of classification accuracy is gradually increasing and finally tends to 100%. However, the increase of symbol length affects the classification accuracy mainly in the case of low SNR. Considering the influence of the computation cost in the classification process, 2048 is chosen as the symbol length in this paper.

**Figure 6:**
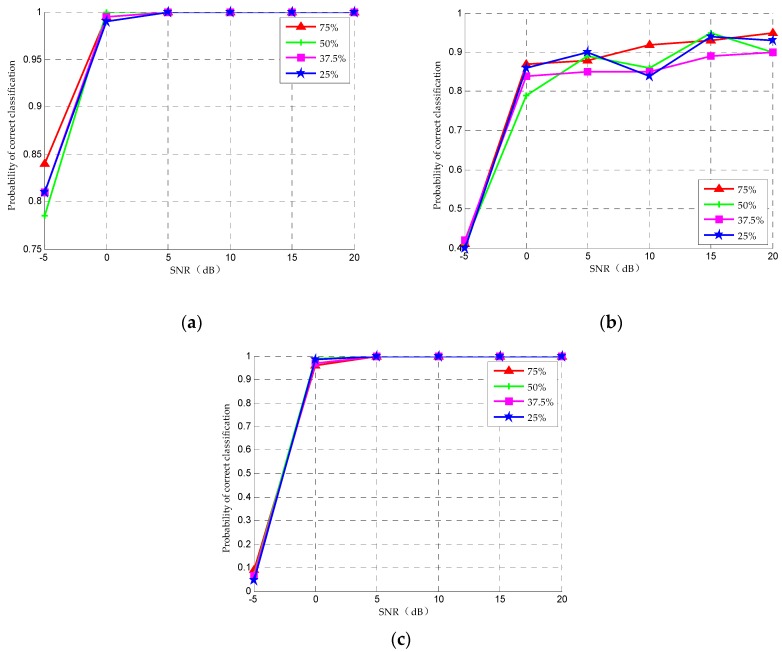
Correct classification rate with different compression rate for (**a**) OOK, (**b**) QPSK and (**c**) 16QAM under different SNRs.

In order to evaluate the impact of different compression ratios on the classification performance, we analyzed the classification accuracy in Figure 6 with the compression ratios of 25%, 37.5%, 50% and 75% for OOK, QPSK and 16QAM signals (as examples) when the symbol length is 2048. As can be seen from Figure 6, with the increase of compression ratio, the classification accuracy increases slightly and finally tends to 100%. The increase of symbol rate has little effect on recognition rate. Therefore, in order to reduce the sampling rate and system complexity as much as possible, 25% is selected as the compression rate value.

In order to optimize the parameters of kernel function and improve the classification accuracy, the grid search method is used. Table 5 shows the optimization results of penalty parameter and kernel parameter of each node of DT–SVM and the classification accuracy of sub-SVM under the RBF kernel function. It can be seen from the table that under different SNR conditions, with the improvement of SNR, the classification accuracy of sub nodes has improved. When the SNR is 0 dB or above, the average classification accuracy has reached 100%.

In order to prove the superiority of this method in recognition accuracy, Table 6 shows the classification accuracy of six different cognitive radio signals using multidimensional HOC, cyclic spectrum and DT–SVM classifier when the kernel function is RBF. In addition, the sizes of training and testing subsets are selected as 80% and 20% of the whole set of eigenvectors. The results show that with the increase of SNR, the classification accuracy of six kinds of signal is improved. When the SNR is 0 dB, the classification accuracy is 100%. It is proved that this method still has high recognition accuracy under low SNR. In addition, a new modulation signal can be introduced to expand the flexibility of the method and shows better compatibility, which will certainly increase the complexity of the algorithm and the classification time of the whole classification system.

## 6. Conclusions

We have proposed a method through simulation to identify the cognitive radio signals based on compressed sensing combined with HOC and cyclic spectrum, which has been proved to perform well in noisy situations. It successfully achieves reconstructing the feature parameters of HOC and cyclic spectrum directly from the sub-Nyquist rate rather than reconstructing the original signal. The simulation results indicate that this method can effectively achieve modulation classification for six kinds of cognitive radio signals with three feature parameters. In this paper, the proposed method is relatively simple and the feature parameters used are few, and they are also less affected by noise. By analyzing the effect of symbol length and compression rate on the classification rate, the classification performance is improved and the classification accuracy can reach 100% when the SNR is 0 dB. This technique utilizes a sampling rate of CS much lower than the Nyquist sampling rate and noise-insensitive feature extraction algorithm, realizes blind classification without any prior information from the transmitter, and has low computational complexity. 

## Figures and Tables

**Table 1:** Theoretical values of higher-order cumulant (HOC) for six wireless signal modulations.

	$\left|C_{4,0}\right|$	$\left|C_{4,1}\right|$	$\left|C_{4,2}\right|$	$\left|C_{6,0}\right|$	$\left|C_{6,3}\right|$	$\left|C_{8,0}\right|$
OOK	2	2	2	16	13	272
DPSK	2	2	2	16	13	272
QPSK	1	0	1	0	4	34
OQPSK	1	0	1	0	4	34
16QAM	0.68	0	0.68	0	2.08	13.9808
64QAM	0.619	0	0.619	0	1.7972	11.5022

**Table 2:** Theoretical values of T1 for six wireless signal modulations.

	OOK,DPSK	QPSK,OQPSK	16QAM	64QAM
T1	136	34	20.56	18.5819

**Table 3:** Theoretical values of HOC after difference between QPSK and OQPSK.

	$\left|C_{d4,0}\right|$	$\left|C_{d4,1}\right|$	$\left|C_{d4,2}\right|$	$\left|C_{d6,3}\right|$	$\left|C_{d8,0}\right|$
QPSK	2	0	2	8	68
OQPSK	2	0	0.89	2	131.4

**Table 4:** Theoretical values of T2 for QPSK and OQPSK.

	QPSK	OQPSK
T2	17	32.85

**Table 5:** The optimization results and identification accuracy of the sub-SVM of each node in DT–SVM.

SNR	SVM-1	SVM-2	SVM-3	SVM-4	SVM-5	AVERAGE
	Acc/% (c,γ)	Acc/% (c,γ)	Acc/% (c_,_γ)	Acc/% (c,γ)	Acc/% (c,γ)	Acc/%
−5 dB	88.33 (2^11.2^,2^13.8^)	100 (2^0^,2^0^)	81.25 (2^0.5^,2^3^)	95 (2^−2.5^,2^15^)	100 (2^0^,2^0^)	92.92
0 dB	100 (2^−8^,2^2^)	100 (2^0^,2^0^)	100 (2^−5^,2^8.5^)	100 (2^−5^,2^7.5^)	100 (2^0^,2^0^)	100
5 dB	100 (2^−8^,2^−2^)	100 (2^0^,2^0^)	100 (2^0^,2^0^)	100 (2^0^,2^0^)	100 (2^0^,2^0^)	100

**Table 6:** The classification accuracy of the cognitive radio signals using multidimensional HOC, cyclic spectrum and DT–SVM classifier.

SNR	Classification Accuracy of Cognitive Radio Signals (%)						
	OOK	DPSK	QPSK	OQPSK	16QAM	64QAM	AVERAGE
−5 dB	72.5	72.5	74.69	74.69	83.25	83.25	76.81
0 dB	100	100	100	100	100	100	100
5 dB	100	100	100	100	100	100	100
10 dB	100	100	100	100	100	100	100
15 dB	100	100	100	100	100	100	100
